# Gum Chewing and Coffee Consumption but not Caffeine Intake Improve Bowel Function after Gastrointestinal Surgery: a Systematic Review and Network Meta-analysis

**DOI:** 10.1007/s11605-023-05702-z

**Published:** 2023-06-05

**Authors:** Stefanie Sinz, René Warschkow, Ignazio Tarantino, Thomas Steffen

**Affiliations:** 1 Department of Surgery, Cantonal Hospital of St. Gallen, Rorschacherstrasse 95, 9007 St. Gallen, Switzerland; 2 Private University of the Principality of Liechtenstein, Triesen, Liechtenstein

**Keywords:** Coffee, Caffeine, Gum chewing, Bowel function, Postoperative ileus, Network meta-analysis, Systematic review, Gastrointestinal surgery, Bowel surgery, Abdominal surgery

## Abstract

**Background:**

Postoperative ileus is common after gastrointestinal surgery. This network meta-analysis aimed to compare the effectiveness of gum chewing and coffee and caffeine intake on ileus-related outcomes.

**Methods:**

A systematic literature review was performed to identify randomized controlled trials (RCTs) comparing noninvasive treatments for ileus after gastrointestinal surgery. The main analyses included random effects network meta-analyses using frequentist methods with simultaneous direct and indirect comparisons of time to first flatus, time to first defecation, and length of stay. Bayesian network meta-analysis using Markov chains was also used.

**Results:**

A total of 32 RCTs comparing 4999 patients were included in this network meta-analysis. Time to flatus was reduced by gum chewing (mean difference compared to control (MD): -11 h, 95% confidence interval (95% CI) − 16 to − 5 h, *P* < 0.001). Time to defecation was reduced by gum chewing and coffee, with MDs of -18 h (95% CI − 23 to − 13 h, *P* < 0.001) and -13 h (95% CI − 24 to − 1 h, *P* < 0.001), respectively. Length of stay was reduced by coffee and gum chewing with MDs of − 1.5 days (95% CI: − 2.5 to − 0.6 days, *P* < 0.001) and − 0.9 days (95% CI: − 1.3 to − 0.4 days, *P* < 0.001), respectively.

**Conclusion:**

Coffee and gum chewing were proven to be effective noninvasive approaches for shortening the postoperative length of hospital stay and time to first defecation, especially in open gastrointestinal surgery; thus these actions should be recommended after gastrointestinal surgery.

**Supplementary Information:**

The online version contains supplementary material available at 10.1007/s11605-023-05702-z.

## Introduction

Postoperative ileus (POI) is a common and significant complication of gastrointestinal surgery. The overall incidence of postoperative ileus varies according to its definition and occurs in approximately 10–30% of patients after abdominal surgery. It is characterized by delayed passage of flatus and defecation, abdominal distension, nausea, vomiting, and the inability to tolerate oral food. Commonly, nasogastric tubes must be inserted, the patient suffers nutritional deficits, and the risk of associated postoperative morbidity increases. ^1–3^

The annual costs of treating POI in the USA are estimated to be at least 750 million dollars. ^4,5^ This is mainly due to the significantly increased duration of hospital stay (approximately 4–9 days) in patients with POI compared to patients with normal recovery of gut function but also due to medication costs, required imaging and personnel costs. ^6^

However, financial burden and POI can increase the patient’s risk of postoperative morbidities such as aspiration pneumonia (0.44–1.4%), anastomotic leakage (up to 5%), or even death (0.9%). ^7–9^

As many factors influence postoperative bowel function, such as anesthesia, postoperative medication, or the surgical procedure per se, numerous different and partially invasive management solutions are discussed.

Gum chewing, caffeine intake, or coffee consumption in the early postoperative period to reduce the occurrence and severity of POI is a promising noninvasive approach that does not contain major side effects. Similar to chewing gum, caffeine and coffee are inexpensive and widely available products that most patients are familiar with and have good experience with, they are well-tolerated interventions in postoperative stages.

Many studies have been conducted to investigate these different approaches with variable outcomes. It has been suggested that gum chewing (GC) and coffee/caffeine intake after surgery may help to recover gastrointestinal function by early stimulation. ^10,11^ The effects of coffee, caffeine, or gum chewing on postoperative bowel movement are debated. Earlier studies reported ambiguous results regarding whether coffee or caffeine had a larger effect; however, there was no difference between coffee and caffeine in terms of an improvement in postoperative bowel movement and a decrease in the length of hospital stay. ^12^

This systematic literature review and network meta-analysis aimed to assess the treatment effect of coffee, caffeine, and gum chewing on postoperative bowel movement in terms of time to first flatus, time to first defecation, and length of stay by simultaneous direct and indirect comparisons. The primary analyses included random effects network meta-analyses using frequentist methods. ^13,14^ Bayesian network meta-analyses served as sensitivity analyses.

## Methods

This systematic review and network meta-analysis was registered in research registry unique identifying number (UIN) of “reviewregistry1541.”

## Data Collection

This review complies with the recommendations of the Cochrane Handbook for Systematic Reviews and Interventions and is reported in line with the PRISMA guidelines and their extension statement for network meta-analyses. ^15–17^ A systematic literature search was performed using PubMed, the Cochrane Library, and Google Scholar. The search string was configured using Boolean operators and medical subject headings (MeSH).

The inclusion criteria were solely randomized controlled studies (RCTs) showing a comparison of at least two treatments measuring at least one of the following outcome parameters: time to first flatus, time to first bowel movement, and length of hospital stay after gastrointestinal surgery. No language restrictions were applied. We excluded reviews, meta-analyses, case reports, letters, comments, and non-RCTs.

A literature search was conducted in the PubMed, Google Scholar, and Cochrane Databases until August 8th, 2022. The search algorithm in PubMed was ((caffeine*) OR (coffein*) OR (coffee) OR (chewing gum) OR (gum chewing) OR (Chew*)) AND ((systematic review) OR (meta-analysis) OR (randomized controlled trial) OR (RCT) OR (randomized)) AND ((bowel function) OR (bowel movement) OR (ileus) OR (postoperative ileus) OR (gastrointestinal motility) OR (recovery)) AND ((colorectal surgery) OR (bowel surgery) OR (colon* surgery) OR (Gastrointestinal surgery) OR (Abdominal surgery) OR (colectomy)).

In the Cochrane database, the search items “chewing gum bowel function surgery,” “coffee bowel function surgery,” and “caffeine bowel function surgery” were used. The Google Scholar search was performed for review articles: ((caffeine*) OR (coffein*) OR (coffee) OR (chewing gum) OR (gum chewing) OR (chew*)) AND ((systematic review) OR (meta-analysis) OR (randomized controlled trial) OR (RCT) OR (randomized)) AND ((bowel function) OR (bowel movement) OR (ileus) OR (postoperative ileus)). Cross-referencing and manual searches of the bibliographies of eligible publications were actively performed to identify further relevant studies for the review. The selection of relevant articles was performed in two stages. First, the titles and abstracts of all retrieved references were screened to determine whether they met the inclusion criteria. Studies considered irrelevant were discarded. Second, we analyzed the full-text articles of each selected abstract. For data extraction, a dedicated predefined spreadsheet was used. Study selection was performed by two researchers with discrepancies resolved through discussion with the involvement of a third researcher. The selection process is illustrated in a PRISMA flow chart. ^18^

## Data Extraction

An Excel sheet was created extracting the type of surgery, measured outcomes, and type of treatment (gum, coffee, or caffeine) from the included studies.

The number of patients in the intervention and control groups and the mean and standard deviation (SD) of the outcome parameters were recorded from text, tables, or figures. Some of the studies reported nonparametric measures instead of the mean and SD for the treatment effects. Hence, the mean and SD for treatment effects were estimated from the mean and SD in the treatment groups. In studies showing only quantiles, the mean and SD were estimated using the Box‒Cox power transformation into the sample mean estimators by Luo et al. and the sample SD estimators by Wan et al. ^19–21^ If possible, missing data were retrieved manually from the figures.

## Publication Bias

Publication bias was separately assessed in pairwise comparisons of the interventions for each outcome parameter because, to the best of the authors’ knowledge, there was no method readily available to examine it in the framework of network meta-analysis. Assessment of publication bias was performed using contour-enhanced funnel plots and significance funnel plots as appropriate. ^22,23^ The latter distinguishes between affirmative studies (i.e., those with a statistically significant and positive estimate) and nonaffirmative studies (i.e., those with a nonsignificant or negative estimate). The ratio eta expresses the likelihood of an affirmative study to be published compared to a nonaffirmative study. ^22^

The risk of bias was assessed by two authors using the ROB-2 tool. ^24^ Consensus was performed with a third author, if necessary.

## Statistical Analysis

Statistical analyses were performed using the R environment version 4.2.1 (http://www.r-project.org) using the recent R libraries “meta,” “netmeta” ^25^, and “gemtc.” Random effects network meta-analyses (NMA) using frequentist methods were the main analyses. They are based on a graph-theoretical method and random effects models. ^14^

Bayesian NMA was performed as a sensitivity analysis. The summary measure for the outcomes was the mean difference (MD) compared to the control.

First, pooled point estimates for each of the outcomes were estimated based on random effects models. Second, pairwise random effects meta-analyses comparing each intervention against the control were performed for all outcomes because none of the included studies compared more than one treatment against the control, hindering such pairwise analyses. These pairwise meta-analyses primarily served to assess statistical heterogeneity to overcome a lack of proven statistical methods to assess statistical heterogeneity in NMA. Statistical heterogeneity was assessed by visual examination of the forest plots, quantified using *I*^2^ and formally tested with Cochran’s Q statistic.

Third, the main analysis, NMA with random effects models based on the frequentist approach, was performed. The network geometry was assessed by network plots. Treatments were ranked using the *P* scores. *P* scores measure the extent of certainty that a treatment is better than another treatment, averaged over all competing treatments. ^26^ This interpretation is comparable to that of the surface under the cumulative ranking curve (SUCRA), which is the rank of a certain treatment within the range of treatments, measured on a scale from 0 (worst) to 1 (best). ^27^ The MD was estimated compared to the control. Due to the spider-like network, net heat plots ^28^ to elaborate inconsistency in the network were not feasible.

Finally, Bayesian NMA was performed as random effects models with Markov chain Monte Carlo (MCMC) simulations based on “Just another Gibbs sampler” (JAGS) ^29,30^ with 25,000 burn-ins, 50,000 inference iterations, and a thinning factor of 10. Point estimates and 95% credible intervals (95% CrI) for the comparison against the control were calculated, and the results were considered statistically significant if zero was not included in the credible intervals. Heterogeneity was assessed by the residual deviance and the deviance information criteria (DIC). Time series, density plots of the samples, and Gelman and Rubin’s plots were used to ensure convergence.

## Results

### Study Selection

**Fig. 1:**
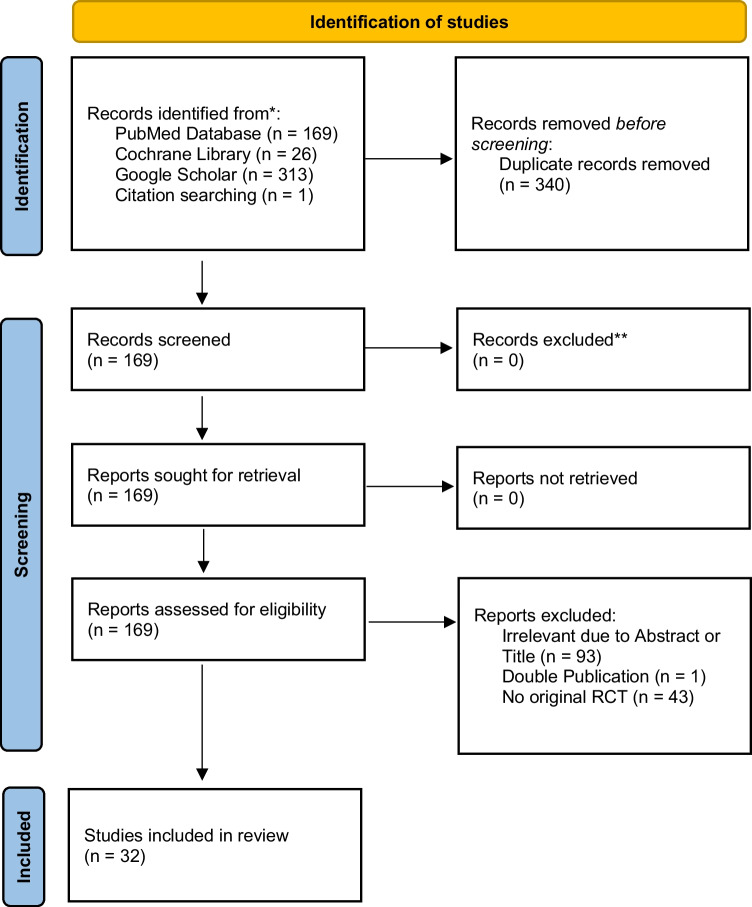
PRISMA flow chart showing the selection of articles for review

A total of 509 studies were identified. After removing 340 duplicates, 169 studies were screened. After excluding noneligible studies (one study was excluded due to double publication, 43 studies were non-RCTs, and 93 studies did not report the required outcome parameters or were not related to gastrointestinal surgery), 32 studies comparing at least one of the treatments against the control and assessing at least one of the outcomes were included in this network meta-analysis (Fig. 1).

### Study Characteristics

The 32 studies included 4999 patients in total. Twenty-six studies reported on gum chewing, and the remaining 6 studies reported on coffee consumption or caffeine intake. Furthermore, 5 out of 32 studies additionally reported on other therapy methods (one studied candy usage, one studied an attention control adhesive patch, one studied the intake of olive oil, one studied the application of a bracelet, and another studied acupuncture). These third arms were not considered in quantitative analyses because they were only assessed once. None of the studies compared more than one treatment against the control (Table 1).

**Table 1:** Systematic review of the studies with descriptions of the type of surgery, measured outcomes, and type of treatment

First author (year)	Total number of included patients	Specification of surgery	Type of surgery	Outcome measured	Treatment
Abbassi (2022)	60	Elective colectomy (RC, LC, PC, SR, RR)	L	FF, FD, LOS	Caffeine
Asao (2022)	19	Elective colectomy not specified	L	FF, FD, LOS	Gum
Atkinson (2016)	402	Elective colorectal resection (TC, RC, LC, RR)	M	FF, FD, LOS	Gum
Bahena-Aponte (2010)	32	Elective LC	O	FF, FD, LOS	Gum
Bhatti (2021)	100	Elective ISC	O	FF, FD, LOS	Gum
Bonventre (2014)	50	Colorectal surgery (RC, LC, RR, HP, TG, GR)	O	FF, FD, LOS	Gum
	25	Colorectal surgery (RC, LC, RR, HP, TG, GR)	O	FF, FD, LOS	Olive oil
Byrne (2018)	158	Elective bowel surgery (RC, LC, TC, SR, RR, Ileostomy closure, SBR)	M	FF, FD, LOS	Gum
Crainic (2009)	42	Elective colectomy (RC, LC, SR, RR)	M	FF, FD	Gum
	19	Elective colectomy (RC, LC, SR, RR)	M	FF, FD	Hard candy
de Leede (2018)	1941	Elective Abdominal surgery (large and small intestine, esophagus/stomach)	M	FF, FD, LOS	Gum
Dulskas (2015)	90	Elective LC	L	FF, FD, LOS	Coffee
Duluklu (2020)	34	Elective LC and/or RR	O	FF, FD, LOS	Gum
Forrester (2014)	31	Elective SR/LC	M	FF, FD, LOS	Gum
	17	Elective SR/LC	M	FF, FD, LOS	Attention control
Ge (2017)	75	Elective TG, GR	L	FF, FD, LOS	Gum
Hasler-Gehrer (2019)	115	Elective RC, LC, SR, RR	L	FF, FD, LOS	Coffee
Hirayama (2006)	24	Elective colon resection (RC, LC, SR, RR)	O	FF, FD	Gum
Kobayashi (2015)	43	Elective LC	O	FF, FD, LOS	Gum
Lim (2013)	157	Elective colorectal surgery (RC, LC, SC, RR)	M	FF, FD, LOS	Gum
Marwah (2012)	100	Elective ISC	O	FF, FD, LOS	Gum
Matros (2006)	43	Elective colectomy (APR, colostomy reversal, RR, RC, LC, PC	O	FF, FD, LOS	Gum
	22	Elective colectomy (APR, colostomy reversal, RR, RC, LC, PC	O	FF, FD, LOS	Bracelet
Müller (2012)	79	Elective colectomy (RC, LC, RR)	M	FF, FD, LOS	Coffee
Ngowe (2010)	46	Emergency appendectomy	O	FF, FD, LOS	Gum
Parnasa (2021)	58	Elective colectomy (RC, LC, SR, PC, RR)	L	FF, FD, LOS	Caffeine
Piric (2015)	59	Elective colon resection (RC, LC, SR)	O	FD, LOS	Coffee
Quah (2006)	38	Elective colorectal cancer surgery (LC, RR, SR, HP, APR)	O	FF, FD, LOS	Gum
Schuster (2006)	34	Elective SR	O	FF, FD, LOS	Gum
Shum (2016)	82	Colorectal resection (RC, LC, SC, TC, RR, APR)	L	FF, FD, LOS	Gum
Topcu (2016)	60	Colorectal surgery (RC, LC, RR)	O	FF, FD, LOS	Gum
van den Heijkant (2015)	112	Elective colorectal surgery (RC, LC, SR, RR)	O	FF, FD, LOS	Gum
Vergara-Fernandez (2016)	64	Elective colon or rectal resection (RC, SR, LC, TC)	M	LOS	Gum
Yang (2017)	379	Elective colorectal cancer resection (TC, LC, RC, RR)	M	FF, FD, LOS	Gum
	186	Elective colorectal cancer resection (TC, LC, RC, RR)	M	FF, FD, LOS	Acupuncture
Yang (2018)	89	Elective proctectomy for rectal cancer	O	FF, FD	Gum
Zaghiyan (2013)	114	Colorectal surgery (RC, LC, RR, SR, APR, IPAA, SC, SBR, TPC, IPAA, ileostomy closure, APR, creation of ileostomy)	M	FF, FD, LOS	Gum

*RC* right hemicolectomy, *LC* left hemicolectomy, *SR* sigmoid resection, *SC* subtotal colectomy, *TC* total colectomy, *PC* partial/segmental colonic resection, *RR* rectum resection, *HP* Hartmann procedure, *TG* total gastrectomy, *GR* gastric resection, *SBR* small bowel resection, *APR* abdominoperineal resection, *TPC* total proctocolectomy, *IPAA* ileal pouch-anal anastomosis, *ISC* intestinal stoma closure, *FF* time to first flatus, *FD* time to first defecation, *LOS* length of stay, *L* laparoscopic, *O* open, *M* mixed laparoscopic and open

### Risk of Bias

Of the included studies, 15 had a low risk of bias, 4 had a low to moderate risk of bias, and 13 had a moderate overall risk of bias. None of the studies showed a high risk of bias. As nearly no intervention was able to blind the patients, nearly all studies therefore had a high risk of performance bias (*n* = 26). Only three studies ^31–33^ were able to blind their patients using either capsules with and without caffeine or by administering coffee with and without caffeine. The remaining coffee consumption groups ^34–36^ were not able to blind their patients, as they compared coffee to placebo (mostly tea or water).

Additionally, missing outcome data (attribution bias) were causative for overall bias (*n* = 14). Reporting bias was only suspected in 4 studies, and 2 studies had high risk (Table 2).

**Table 2:**
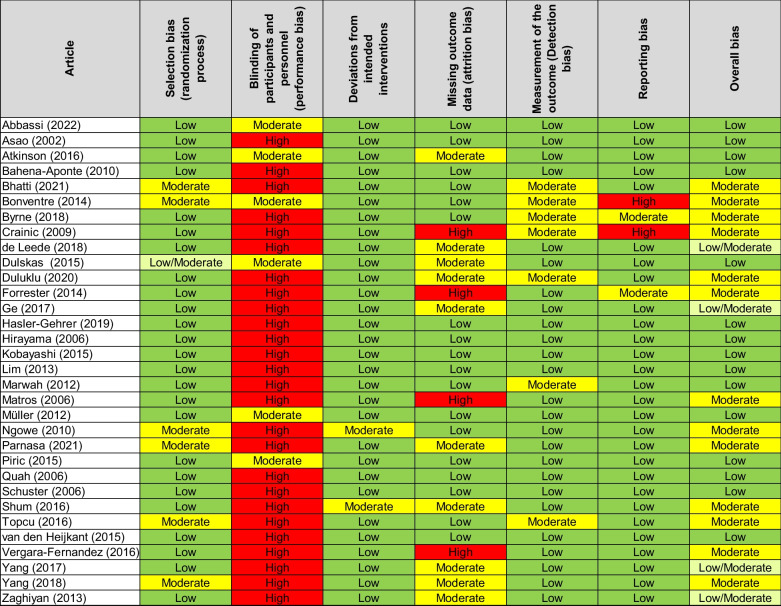
Risk of bias

### Assessment of Publication Bias

The contour-enhanced funnel plots for gum chewing, coffee consumption, and caffeine intake in first flatus, first defecation, and length of hospital stay were fairly robust to publication bias and did not indicate the presence of a strong publication bias (Supplementary Figure S1). Network graphs for the presence of publication bias including time to first flatus, time to first defecation, and discharge were compared to controls. Each node represents a treatment. The thickness of each line indicates the number of studies comparing the two treatments. The control group constituted the center of the spider-like network as there was no study with a head-to-head comparison between the treatments (Supplementary Figure S2).

### Meta-analysis

Pooled point estimates for time to first flatus were 50.8 h (95% CI: 46.0 to 55.6 h), for time to first defecation 69.4 h (95% CI: 62.0 to 76.7 h), and for length of hospital stay 6.4 days (95% CI: 5.7 to 7.1 days) independent of the treatment.

**Fig. 2:**
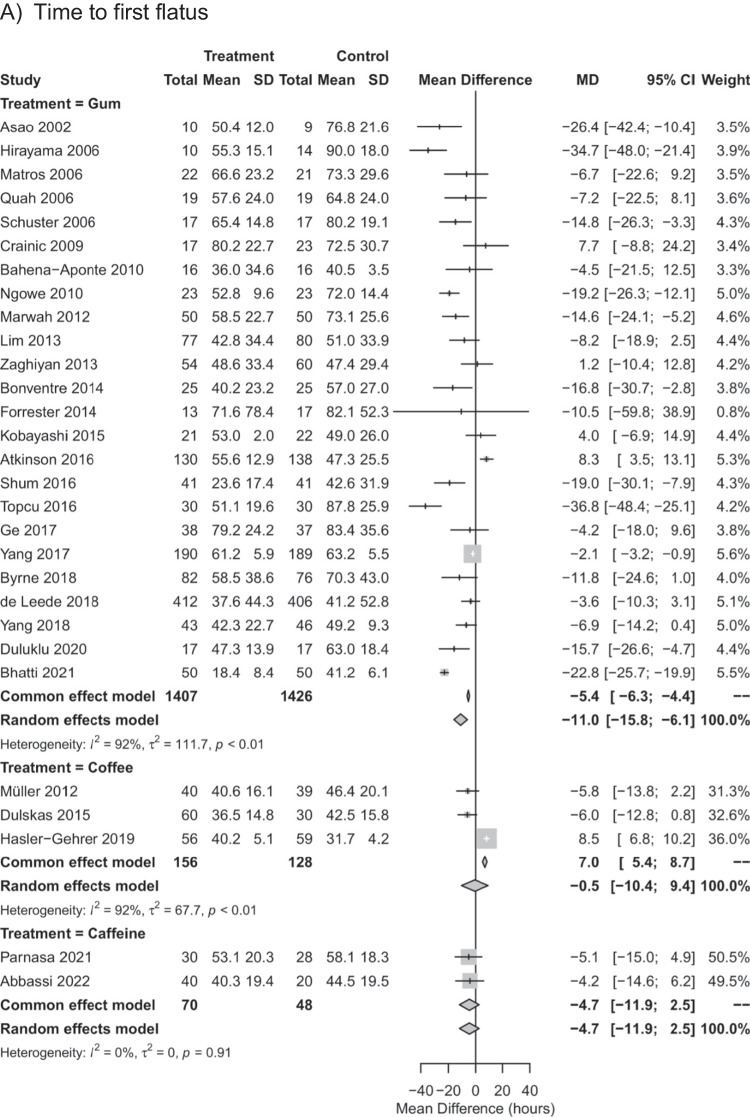

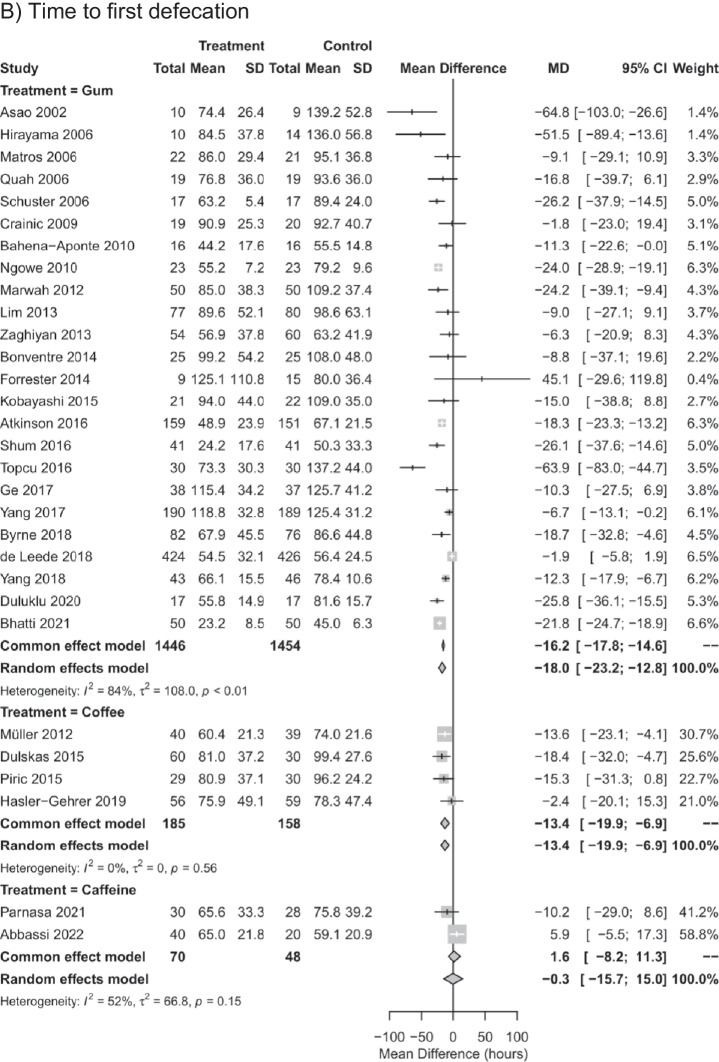

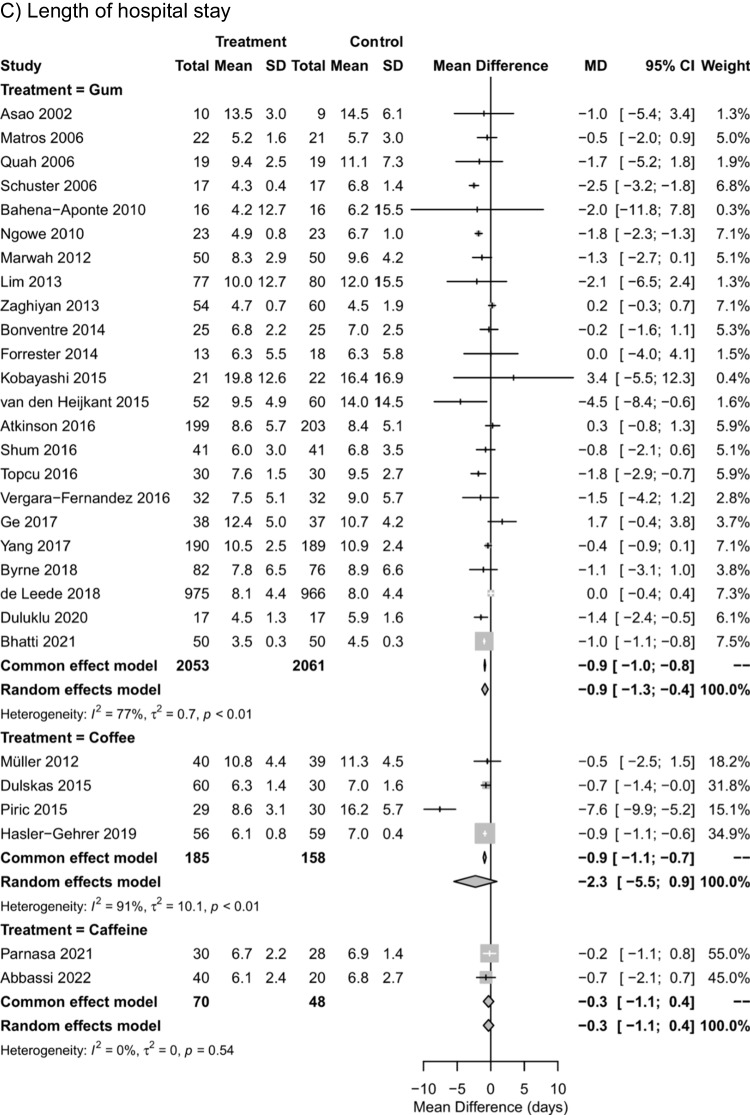
Forest plot for univariable random-effects meta-analyses of the mean difference in pairwise comparisons against control for time to first flatus (**A**), time to first defecation (**B**), and length of hospital stay (**C**)

Pairwise meta-analyses comparing each intervention against control for time to first flatus, time to first defecation, and length of hospital stay (Fig. 2) indicated substantial to considerable statistical heterogeneity for gum chewing by visual inspection of the forest plot and by *I*^2^ ranging from 77 to 92%. For coffee intake, considerable statistical heterogeneity was observed for time to first flatus and for length of hospital stay (*I*^2^ = 91% and *I*^2^ = 92%) but not for time to first defecation (*I*^2^ = 0%). For caffeine, moderate statistical heterogeneity was observed for time to first defecation (*I*^2^ = 52%). In pairwise meta-analyses, gum chewing reduced the time to first flatus, time to first defecation, and length of hospital stay by MD of -11.0 h (95% CI: -15.8 to -6.1 h), -18.0 h (95% CI: -23.2 to -12.8 h), and -0.9 days (95% CI: 1.3 to -0.4 days), respectively, compared to the control. Coffee intake reduced the time to first defecation by a MD of -13.4 h (95% CI: -19.9 to -6.9 h), but not the time to first flatus (MD of -0.5 h with 95% CI: -10.4 to 9.4 h) or length of hospital stay (MD of -2.3 days with 95% CI: -5.5 to 0.9 days). Caffeine intake had no effect on time to first flatus (MD of -4.7 h with 95% CI: -11.9 to 2.5 h), time to first defecation (MD of -0.3 h with 95% CI: -15.7 to 15.0 h) or length of hospital stay (MD of -0.3 days with 95% CI: -1.1 to 0.4 days).

### Network Meta-analysis

The network graphs revealed spider-like nets and no closed loops due to the lack of studies with a head-to-head comparison between the treatments. For time to first defecation, 25, 4, and 2 studies compared gum chewing, coffee, and caffeine intake, respectively, with control (Table 1).

**Fig. 3:**
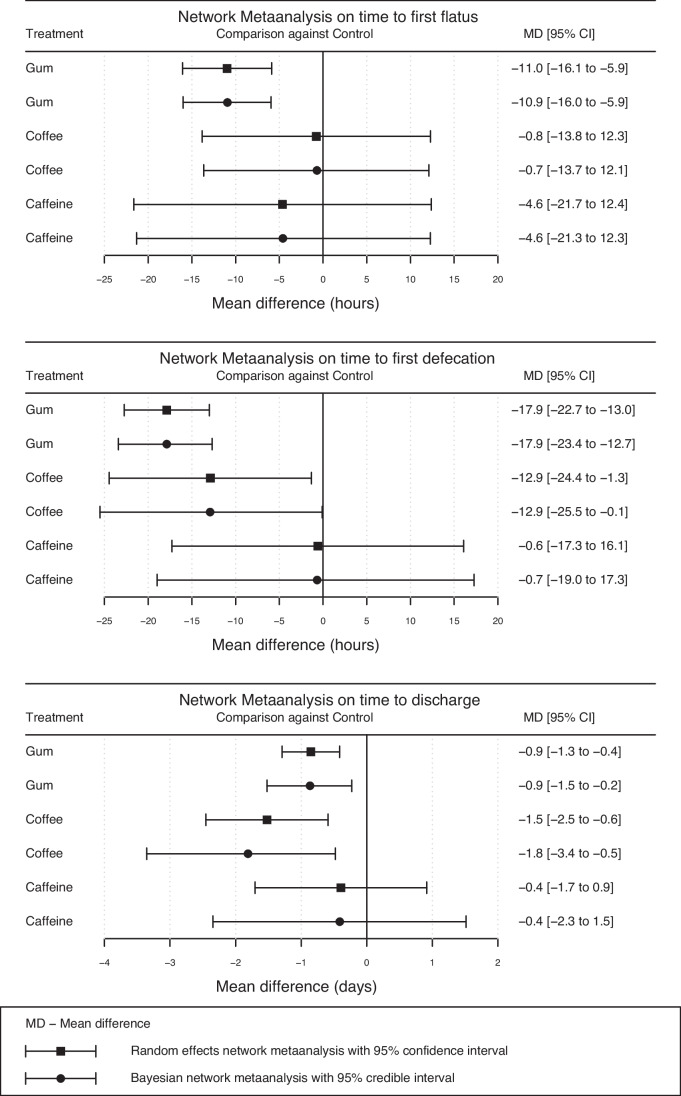
Summary plot for point estimates and their corresponding 95% confidence and credible intervals in the frequentist and Bayesian network meta-analyses

Substantial statistical heterogeneity was confirmed for all three outcomes (*P* < 0.001), especially for gum chewing. Gum chewing was ranked best for time to first flatus and first defecation whereas coffee consumption was ranked best for length of hospital stay. The *P* values for gum chewing, coffee consumption, and caffeine intake for time to first flatus were 0.89, 0.33, and 0.53, respectively; the *P* values for time to first defecation were 0.92, 0.69, and 0.22, respectively; and the *P* values for length of hospital stay were 0.61, 0.94, and 0.35, respectively. The time to first flatus was reduced by gum chewing with a MD of -11 h, (95% CI − 16 to − 5 h, *P* < 0.001) (Fig. 3). Time to first defecation was reduced by gum chewing with a MD of -18 h (95% CI − 23 to − 13 h, *P* < 0.001) and by coffee with a MD -13 h (95% CI − 24 to − 1 h, *P* < 0.001). Length of stay was reduced by coffee and gum chewing with MDs of − 1.5 days (95% Cl: − 2.5 to − 0.6 days, *P* < 0.001) and − 0.9 days (95% CI: − 1.3 to − 0.4 days, *P* < 0.001). Coffee was not superior to gum chewing in terms of length of hospital stay, with a MD of − 0.7 days (95% CI: − 1.7 to 0.4 days, *P* = 0.203). No significant effect was observed for caffeine. These results were confirmed by sensitivity analysis with Bayesian NMA with quite similar point estimates and 95% CI (Fig. 3).

**Fig. 4:**
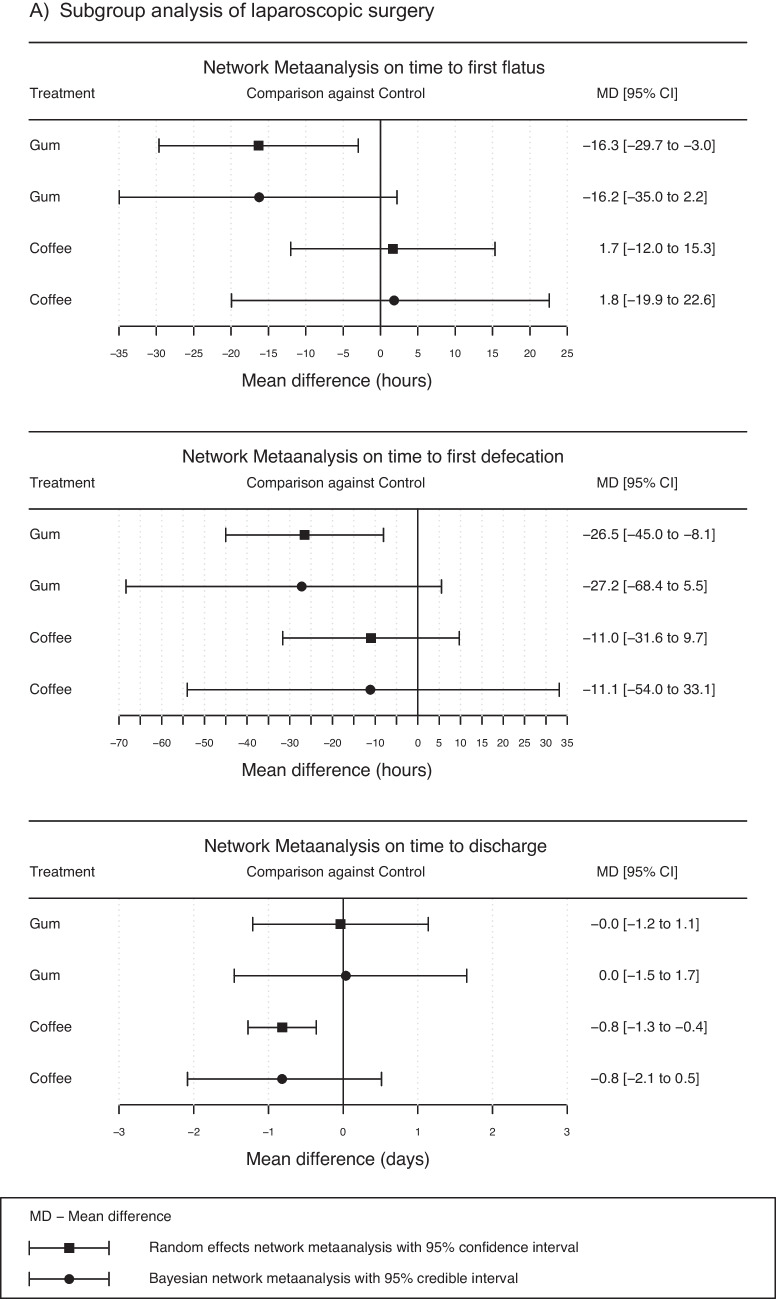

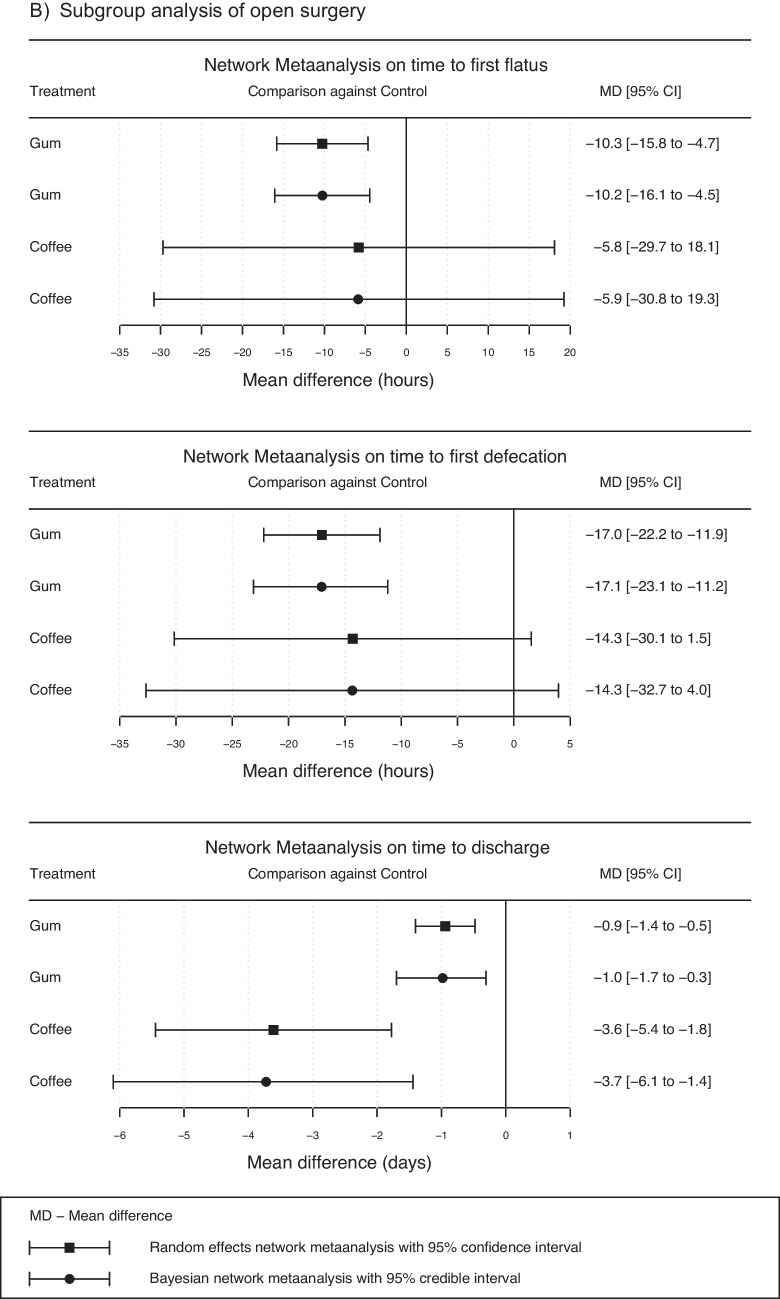
Subgroup analysis for point estimates and their corresponding 95% confidence and credible intervals in random effects and Bayesian network meta-analysis in laparoscopic (**A**) and open (**B**) gastrointestinal surgery

The results of two subgroup analyses after laparoscopic and open gastrointestinal surgery are summarized in Figs. 4a and b to elaborate possible differences in gum chewing, coffee consumption, and caffeine intake**.** Studies with mixed laparoscopic and open surgeries were added to the open group, as there were either many conversions in those studies or missing specifications in the percentage of conversion rates. The subgroup analyses of gum chewing and coffee consumption showed a significant reduction in time to discharge in the open surgery group (95% CI (gum): -1.7 to -0.3 days, respectively 95% CI (coffee): -6.1 to -1.4 days). In the laparoscopic group, there was no significant effect on time to discharge (95% CI (gum): -1.2 to 1.1, 95% CI (coffee): -2.1 to 0.5).

The time to first defecation in the gum-chewing open group (95% CI: -23.1 to 11.2 h) showed a significant reduction but did not carry weight in the time to discharge in this group.

Attempts to further elucidate the within-group heterogeneity by mixed effects meta-regression analyses using year of publication and laparoscopic versus open surgery as moderator variables did not succeed in relevantly decreasing the within-group heterogeneity. The *I*^2^ in the meta-regressions remained high and ranged from 51 to 84% compared to 77 to 92% in pairwise meta-analyses with laparoscopic versus open surgery as a significant moderator variable for all outcomes comparing gum chewing with control.

## Discussion

This systematic review found clinically relevant and statistically significant effects on postoperative ileus after gastrointestinal surgery for gum chewing and coffee consumption but not for caffeine intake. These findings were confirmed by pairwise meta-analysis, random effects NMA using frequentist methods, and Bayesian network meta-analyses. Gum chewing was associated with an improvement in all assessed outcomes. Coffee consumption shortened the time to first defecation and length of hospital stay but not the time to first flatus. A numerically stronger effect for coffee consumption compared to gum chewing on the length of hospital stay could not be proven. The grade of evidence of this review was relevantly impaired by statistical heterogeneity, more pronounced for gum chewing than for coffee consumption.

Subgroup analysis in laparoscopic and open surgery confirmed a significant reduction in time to discharge in open surgery. In the subgroup analysis with laparoscopic surgery, the effect of gum could not be confirmed. One probable explanation might be the introduction of enhanced recovery programs in laparoscopic surgery. As the laparoscopic approach reduces abdominal wall trauma and the resultant milieu of inflammatory, neurohumoral, and pain responses, the recovery process might be accelerated. ^37,38^

For the research question at hand, length of hospital stay could be seen as the most objective measure and the easiest one to record. For coffee consumption and gum chewing, clinically relevant shortening of 1.5 days and 0.9 days was observed. In contrast, no effect was observed in the caffeine group, thus confirming recent research reporting no effect of caffeine on POI in a homogeneous RCT assessing exclusively laparoscopic colectomy. ^31^

Coffee consumption and gum chewing reduce the costs associated with the length of hospital stay. As they are inexpensive products that many people are familiar with, their use is widely accepted by patients. Watanabe et al. and Eamudomkarn et al. described similar findings, which were a shortening of LOS with postoperative coffee consumption, especially with the increasing complexity of the surgical procedure. ^39,40^ In addition, a shortening of the average length of hospital stay leads to a reduction in health care costs. ^41^

Physiologically the gastrointestinal system is stimulated by meals with high calories, acidity, or osmolarity or due to its volume. In the case of chewing gum, coffee consumption, and caffeine intake, their ingredients must exert biochemical effects, as they are almost free of calories and have low osmolality. ^33^

Coffee and its bioactive compounds are suggested to influence the gastrointestinal mucosa (permeability, secretion, and proliferation), the gut wall (and its neural and nonneural components), and the brain–gut axis. ^42^ Coffee consumption also induces cholecystokinin release, gallbladder contraction ^43^, and a gastrocolic response with increased colonic motility. ^44,45^ As different ingredients in coffee and caffeine exist, the different outcomes in the length of stay, time to first flatus, and time to the first defecation are explained by the different ingredients, such as melanoidins or chlorogenic acid. ^46^ These different compositions are due to the different coffee bean species as well as the roasting process, which leads to a strong variation in composition. ^46,47^

Chlorogenic acid is supposed to have an anti-inflammatory effect by inhibiting the production of interleukin-6 and tumor necrosis factor alpha and therefore reduces inflammation and pain with the improvement of gastrointestinal recovery, whereas melanoidins partially behave as dietary fiber, as shown in in vivo experiments, and have the ability to influence the contractility of gastric smooth muscles by activating cholinergic receptors. ^46,48^

Considering the physiological mechanism by which gum chewing improves bowel function after surgery, it is assumed that gum chewing activates the cephalic-vagal pathway via the parasympathetic nervous system. This stimulates intestinal myoelectric activity and bowel motility by counteracting the activation of the gastrointestinal μ-opioid receptor. It also seems to release gastrointestinal hormones and increase the secretion of saliva and pancreatic juice and therefore stimulates bowel movement. ^4,49,50^ Short et al. found some evidence that gum chewing may influence the digestive system to recover, especially bowel sounds and decrease the length of stay, but the involved studies were less reliable due to poor quality and a lack of description of methodology and allocation. ^51^

In contrast, de Leede et al. described no evidence of gum chewing in the postoperative care pathway to reduce the time to bowel recovery or length of stay in their RCT, which might be due to the heterogeneity of the frequency and duration of gum chewing. ^52^

There are several limitations of the present study, particularly with respect to the blinding of the participants. First, the participants in the gum-chewing group were not blinded due to its impracticability, which can lead to a high risk of performance bias. In the caffeine group, only two studies were able to blind their patients by using capsules with and without caffeine. The coffee consumption group contained only one study that blinded the participants by administering coffee with and without caffeine. As coffee compounds vary by region, bean type, and roasting method, the effect of coffee consumption may not be generalizable to all populations, which limits the validity of the RCT analysis. The characteristics of regular and nonregular coffee drinkers were not reported in the studies. Additionally, the dose‒response relationship between coffee and caffeine consumption is unknown, as it was not evaluated. Furthermore, the relevant in-group heterogeneity could not be explained, further limiting the generalizability of the present study, particularly for treatment with gum chewing, for which a significant treatment effect could only be proven after open surgery but not after laparoscopic surgery. Finally, the small number of included studies has also to be mentioned. Only four, respectively two studies reported on coffee consumption and caffeine intake. Given the small number of studies with a quite small included number of participants, the power to differentiate between these two treatments has to be considered strongly limited. Further evidence is needed to discriminate between these two treatments with certainty.

The main strength of the present investigation is the fact that only RCTs were included in the study. This is the first network meta-analysis comparing chewing gum, coffee consumption, and caffeine intake. Despite relevant heterogeneity in the univariable meta-analyses, the random effects NMA using frequentist methods and the Bayesian analyses gave quite similar results. The present study demonstrated that time to first flatus and defecation should be considered surrogate parameters in the gum chewing and coffee consumption groups for the length of hospital stay, as there were significant reductions in the univariate analysis but not in the network analysis.

## Conclusion

Coffee and gum chewing were proven to be effective and noninvasive approaches for shortening the postoperative length of hospital stay and time to first defecation, especially after open gastrointestinal surgery; thus, these actions should be recommended after gastrointestinal surgery.

## Supplementary Information

Below is the link to the electronic supplementary material.

Supplementary file1 (DOCX 455 KB)

## Data Availability

The data that support the findings of this study are available from the corresponding author upon reasonable request.
