# Supplementary material for: Gum Chewing and Coffee Consumption but not Caffeine Intake Improve Bowel Function after Gastrointestinal Surgery: a Systematic Review and Network Meta-analysis
Source: J Gastrointest Surg. 2023 Jun 5;27(8):1730–45. doi: 10.1007/s11605-023-05702-z (PMC10412511; doi:10.1007/s11605-023-05702-z)
Supplement: Supplementary file 1 — Supplementary file1 (DOCX 455 KB) [file 11605_2023_5702_MOESM1_ESM.docx]

**Supplementary Figures**

**Supplementary Figure 1:** Contour-enhanced funnel plots for assessment of publication bias in gum chewing (panels A to C), in coffee consumption (panels D to F), and caffeine intake (panels G to I) for first flatus (A, D, G), first defecation (panels B, E, H), and length of hospital stay (panels C, F, I).

**Supplementary Figure 2**: Network graphs for time to first flatus, time to first defecation and discharge.

**Supplementary Figure 1:** Contour-enhanced funnel plots for assessment of publication bias in gum chewing (panels A to C), in coffee consumption (panels D to F), and caffeine intake (panels G to I) for first flatus (A, D, G), first defecation (panels B, E, H), and length of hospital stay (panels C, F, I).

Funnel plot in caffeine intake

Funnel plot in coffee consumption

Funnel plot in gum chewing


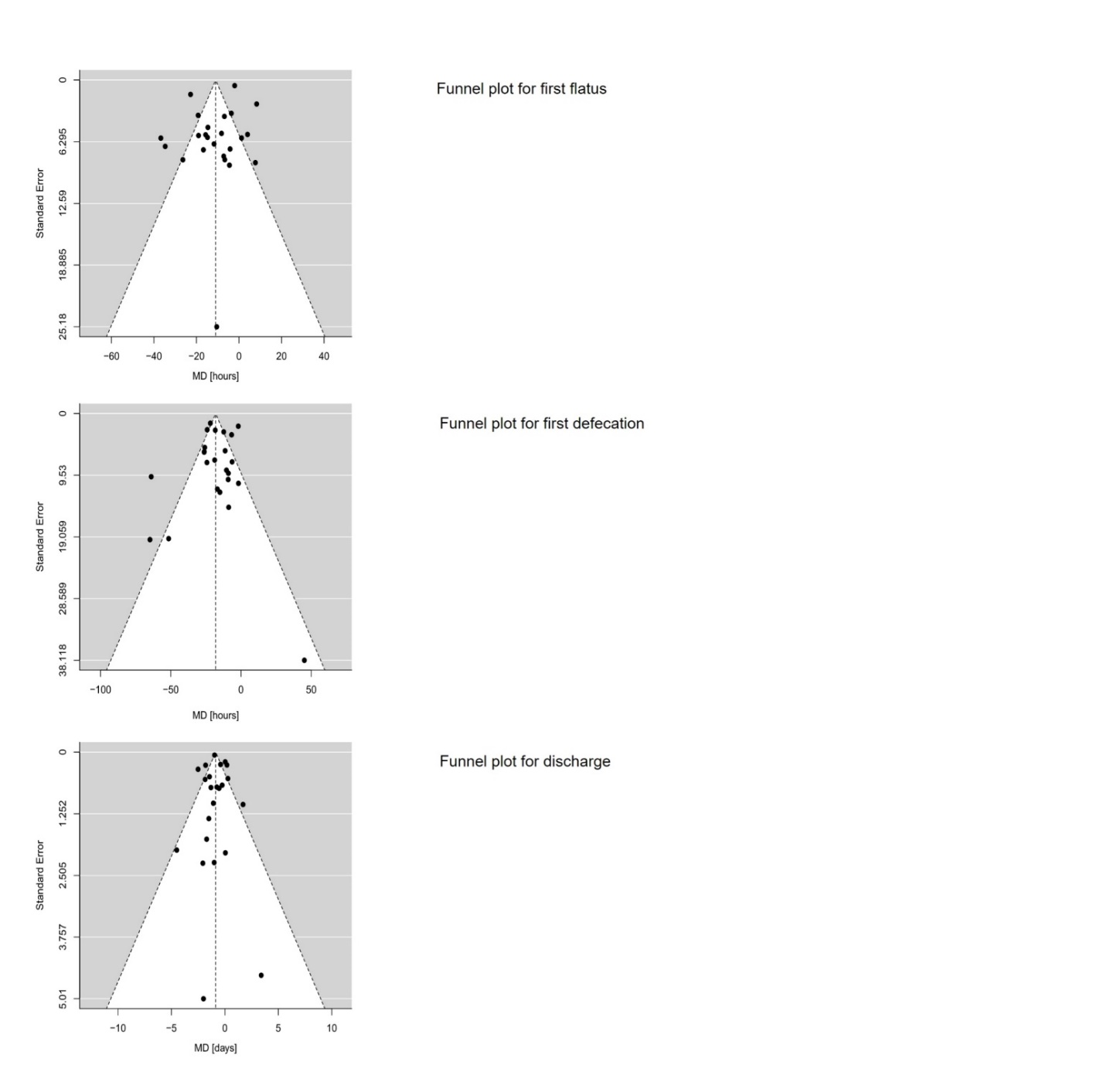

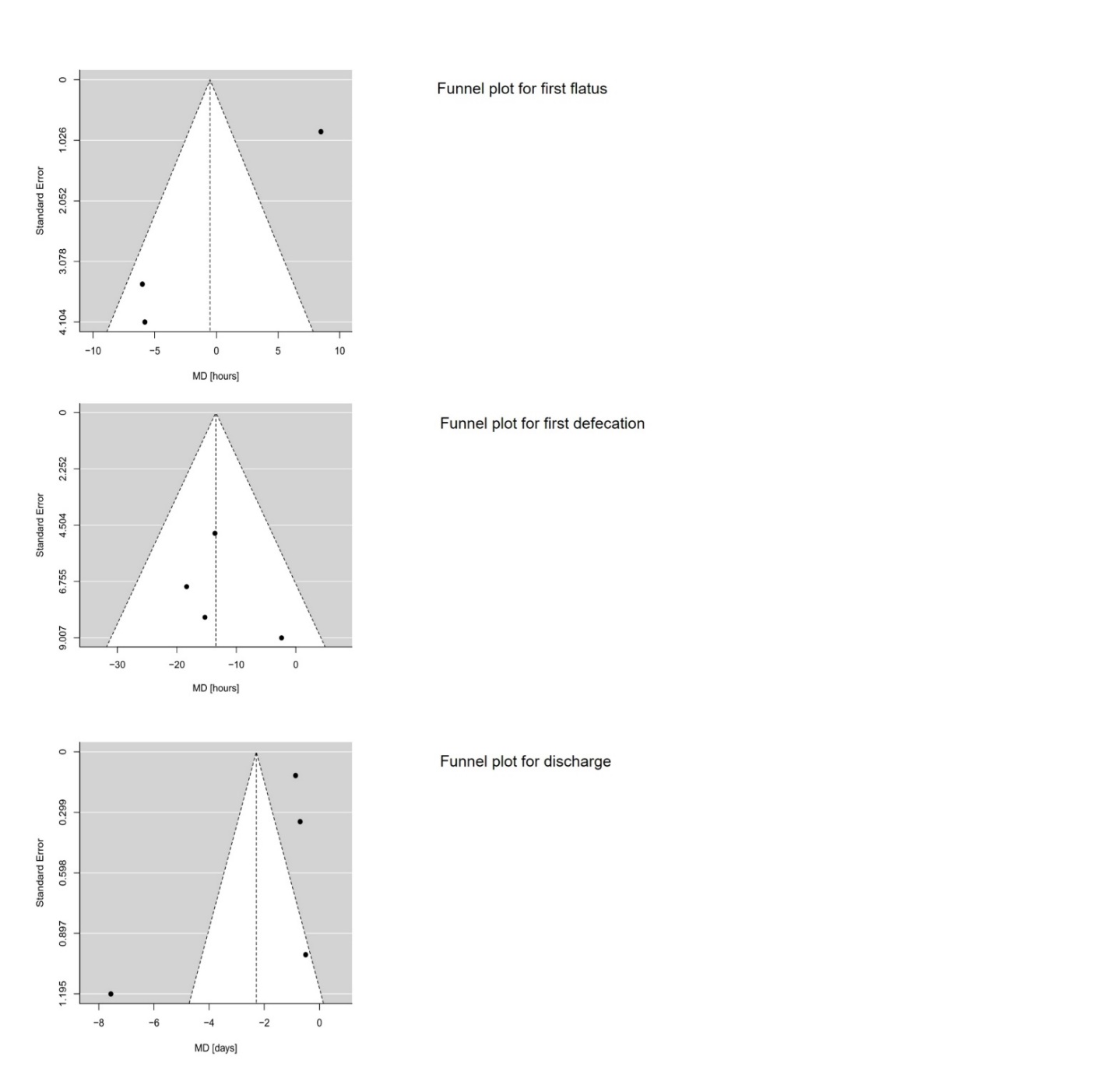

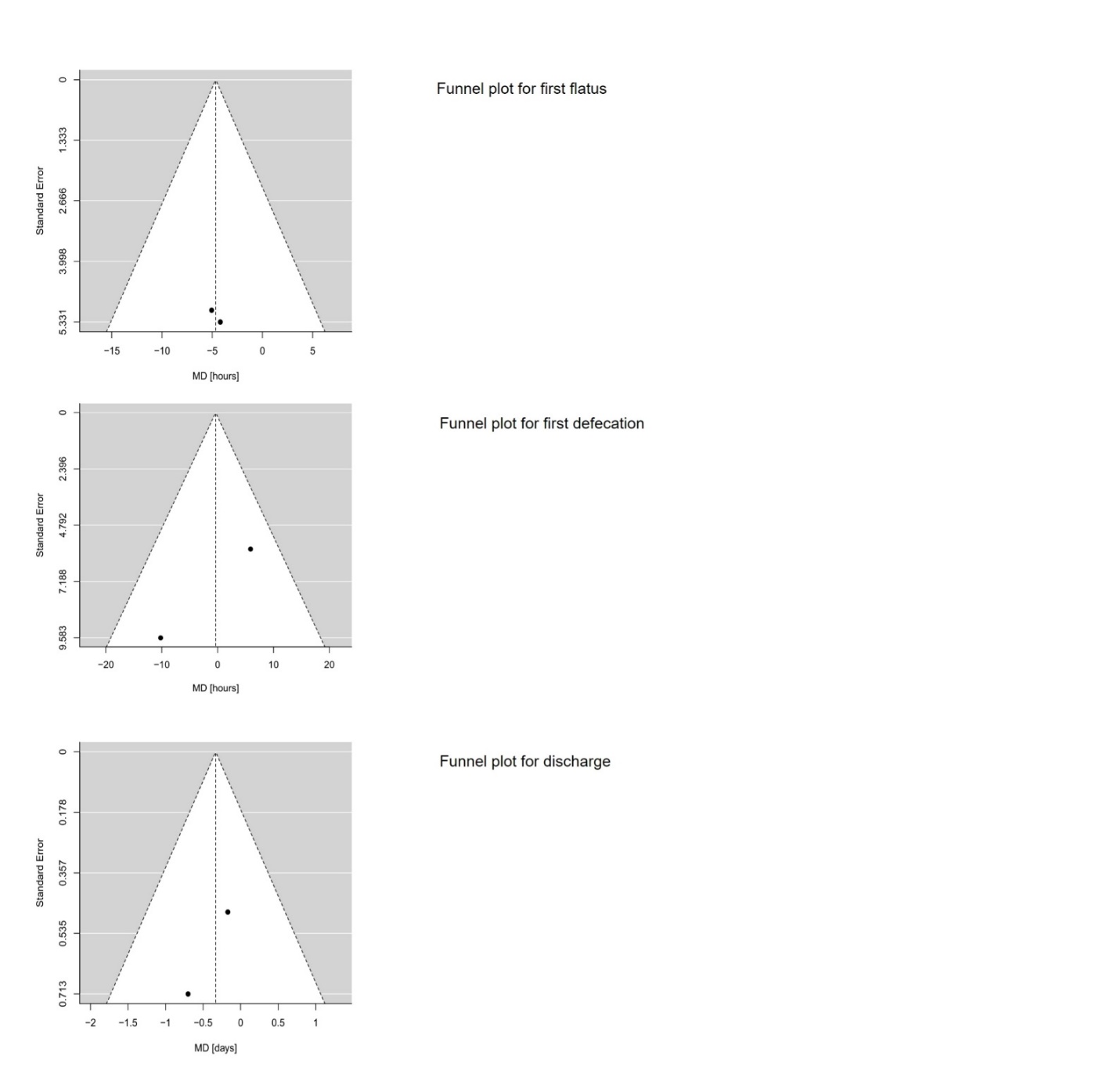


H)

C)

F)

I)

G)

E)

D)

A)

B)

The treatment effect is plotted on the horizontal axis, and the standard error is plotted on the vertical axis. The vertical line represents the summary estimated using fixed effects meta-analysis. Two diagonal lines represent 95% confidence limits around the summary effect for each standard error on the vertical axis. These show the expected distribution of studies in the absence of heterogeneity or of selection bias. In the absence of heterogeneity, 95% of the studies should lie within the funnel defined by these diagonal lines.

**Supplementary Figure 2**: Network graphs for time to first flatus, time to first defecation and discharge.


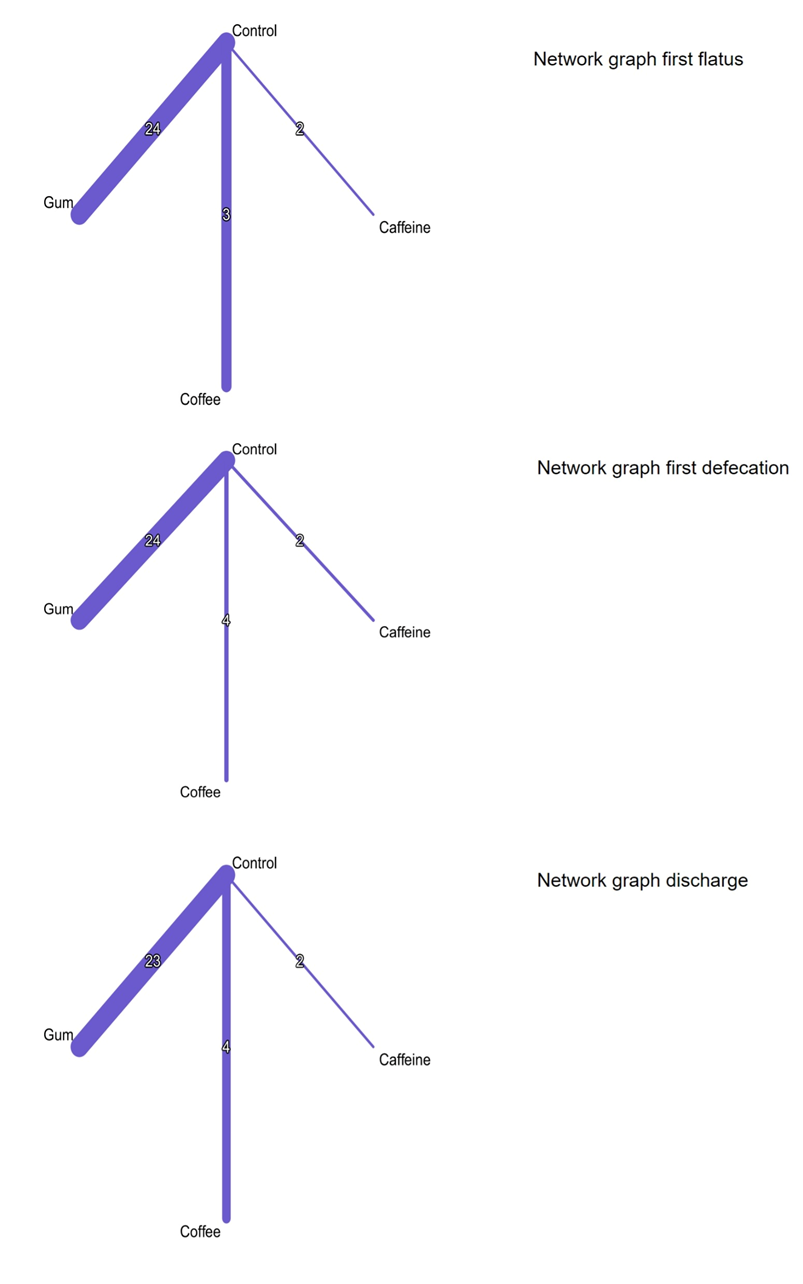


Each node represents a treatment. The thickness of each line indicates the number of studies comparing the two treatments. The three treatment modalities were compared to the control, the latter constituting the center of the spider-like network because there was no study with a head-to-head comparison between the treatments.
